# Theorising Health System Resilience and the Role of Government Policy- Challenges and Future Directions: Comment on "Government Actions and Their Relation to Resilience in Healthcare During the COVID-19 Pandemic in New South Wales, Australia and Ontario, Canada"

**DOI:** 10.34172/ijhpm.2022.7364

**Published:** 2022-07-03

**Authors:** Janet E. Anderson

**Affiliations:** Department of Anaesthesiology and Perioperative Medicine, Faculty of Medicine, Nursing and Health Sciences, Monash University, Melbourne, VIC, Australia.

**Keywords:** Resilient Healthcare, COVID-19, Multi-level Resilience, Health System Resilience, Government Policy

## Abstract

Resilient healthcare (RHC) emphasises the importance of adaptive capacity to respond to unanticipated crises such as the global coronavirus disease 2019 (COVID-19) pandemic but there are few examples of RHC research focusing on the decisions taken by macro level policy makers. The Smaggus et al paper analyses the actions of two governments in Canada and Australia as described in media releases from a resilience perspective. The paper clearly articulates the need for conceptual clarity when analysing system resilience, and integrates three theoretical perspectives to understand the types of government responses and how they were related to resilience. The paper makes a valuable contribution to the developing RHC evidence base, but challenges remain in identifying conceptual frameworks, researching macro level resilience, including identifying and accessing reliable macro level data sources, analysing interactions between macro, meso and micro system levels, and understanding how resilience manifests at different temporal and spatial scales.

## Introduction

 Over the past decade the foundational principles of a new approach to studying and improving healthcare systems have been developed. Building on Resilience Engineering, which addresses safety in a range of sociotechnical domains, resilient healthcare (RHC) has generated widespread interest and optimism about the potential for improving healthcare quality and safety. RHC views healthcare as a complex adaptive system within which the actions of multiple actors operating at different levels of the system and at different times and scales of action interact to create outcomes.^1^ However, there are limitations which must be addressed for the field to move forward, including lack of conceptual clarity,^2^ limited understanding of the complexity of resilience at the macro level, and lack of methodological guidance for studying the complex interactions between system components and levels. Thus, our understanding of healthcare system resilience is limited.

 The paper by Smaggus et al addresses some of these shortcomings and represents an advance in the RHC knowledge base. The coronavirus disease 2019 (COVID-19) pandemic created the need for extensive adaptive actions in healthcare systems worldwide, and so provides a good opportunity for studying system resilience. In this commentary I will consider how Smaggus et al have addressed some of these limitations and highlight some of the challenges that remain, including developing clear conceptual and theoretical frameworks, limited understanding of how actions at the macro level affect system resilience, and research methods for understanding the complex multilevel interactions that create or constrain resilience.

## Conceptual and Theoretical Clarity

 In general, system resilience refers to the ability of a complex system to flexibly adapt to the conditions it is operating in. However, resilience has been interpreted differently by researchers, and a range of resilience definitions, concepts and mechanisms have been proposed. It is only with time and research that a consensus about the most important and useful concepts emerges. Smaggus et al^3^ insightfully discuss Woods’^4^ conceptualisation of four types of resilience; recovery/rebound, robustness, graceful extensibility and sustained adaptability. They highlight the importance of moving beyond reactive resilience to proactively plan and invest in adaptive capacity so that unforeseen crises can be effectively managed. Distinguishing the meaning of related but distinct concepts such as these is important for articulating the goals and benefits of proposed actions. It facilitates better evaluation of the available options for action, better decision making, and better learning about what worked and what did not. An important contribution of the Smaggus et al^3^ study is how clearly the authors articulate the need for conceptual clarity and the consequences of confusion. They have highlighted how the principles of RHC could inform policy by providing a framework within which to consider and evaluate different courses of action, and by focusing attention on neglected aspects of resilience such as ensuring sustained resilience over time.

 Smaggus et al^3^ integrated several resilience theoretical frameworks to guide their work, illustrating how theory can inform research and highlighting examples of helpful and usable theoretical frameworks. First, they used the resilience potentials^5^ to understand how actions can help or hinder health system resilience through anticipation, monitoring, responding and learning. Second, they drew on the Resilience Attributes Framework^6^ which integrated the CARE resilience model^7^ concepts of adaptation driven by demand-capacity misalignments, the importance of creating the potential for resilient performance through supporting anticipating, monitoring, responding and learning,^5^ and the Moments of Resilience model^8^ to conceptualise how actions at various system levels, times and spatial scales can be defined and operationalised for study. The Moments of Resilience framework proposes three temporal-spatial scales of action – situated, structural and systemic. At the systemic level resilient actions are focused on reformulating how resources and practices are produced over an entire system. In response to the systemic shock of a pandemic, this is an accurate description of the task facing governments and regulatory bodies.

 Defining and applying these concepts and frameworks, as Smaggus et al have done, enables deeper understanding of the implications of government actions for resilience and highlights which aspects of resilient performance were emphasised and which were neglected. The work has also highlighted the need to draw on a range of overlapping frameworks in the absence of a parsimonious, integrated framework of system resilience. This is potentially confusing and may increase the difficulty of communicating the concepts to a wider audience. Further refining of theoretical frameworks based on empirical work is needed to build understanding and accelerate the uptake of RHC principles. The pandemic has clearly highlighted the urgency of this need.

## Understanding Macro Level Influences on System Resilience

 Understanding resilience at micro and meso levels is not sufficient to understand system resilience. Governments and regulatory agencies make decisions that support, hinder, or constrain resilience, but as others have highlighted, there are few macro level analyses of resilience^9^ One exception is an analysis of Norwegian quality and safety regulation using an RHC perspective^10^ which highlighted the ambiguity of regulatory approaches to resilience but concluded that regulation and resilience are not incompatible, despite the RHC emphasis on the limitations of rules and standardisation. Regulation can in theory support safe adaptation within a regulatory framework but determining the parameters to achieve this is not clear. Smaggus et al have focused on the macro level to examine adaptive responses to the pandemic in two countries and have expanded the scope of RHC research to different system levels and to international comparison. They analysed media releases to determine which actions were taken and how they may have helped or hindered healthcare system resilience.

 Their analysis showed that governments concentrated on actions to ensure recovery and robustness and paid less attention to graceful extensibility and sustained adaptation. They found more actions in the responding category than in monitoring, anticipating or learning. As the authors point out, this may simply imply that governments highlighted these actions to their constituents because they assumed that evidence of responding would be perceived positively. The balance between these different types of communications may well have shifted during the pandemic and outside the period of the data collection as different problems emerged (such as the need to anticipate future developments) and received attention. Applying RHC concepts and theories to analyse government actions during a crisis is helpful for organising our understanding of the responses and highlights the importance of continuing to focus research efforts on actions and developments at the macro level.

 However, the macro level is complex and multi layered, including regulatory and financial mechanisms, competition, professional regulation technology, and geographical/population factors.^11^ Many bodies with overlapping and sometimes competing goals determine the context within which provider organisations must function. Many studies, including Smaggus’ study do not map the macro context in detail, precluding analyses of the interaction between macro level organisations, and between the macro and meso/micro levels of the system. Understanding such interactions will likely be important for understanding system resilience.

 As the Moments of Resilience model reminds us, resilience occurs at different spatial and temporal scales, and an analysis of media releases does not enable scrutiny of previous government actions that may have enabled or constrained the capacity for adaptation during the pandemic. Organisations need a margin of manoeuvrability^12^ in order to be able to adapt and respond appropriately to crises. A resource-constrained, stretched organisation is unlikely to have the means or slack resources^13^ required to adapt to pressures and problems quickly when an unexpected crisis develops. A brief overview of actions taken by the UK government during the pandemic shows that resources and processes were rapidly reconfigured, including providing extra clinical capacity through building major new intensive care units throughout the country, reconfiguring existing space, enabling students to enter practice earlier than usual, enabling retirees to return to practice, relaxing staffing levels to allow nursing staff to monitor multiple patients, cancelling and suspending some treatments and procedures, introducing telehealth consultations, and abolishing previously implemented targets such as the emergency department four hour rule. Although there were many actions initiated, some, such as building new intensive care units were only partly successful because there was little margin for manoeuvrability, especially for staffing the units. Others, such as cancelling treatments and increasing staff workloads, although successful at the time, will have negative effects in the future on patients who need treatment, and staff who experience burnout. The provision of a margin of manoeuvrability has the potential to reduce such negative effects because it provides a resource buffer that can be drawn on when needed.

 Understanding resilience at the macro level of healthcare systems will require careful analysis of these contextual features of the system and the ability to tease out the interactions between system levels and outcomes at different spatial and temporal scales.

## Research Challenges

 Smaggus et al have highlighted the difficulty of researching how government actions affect healthcare system resilience. As they explain, media releases are freely available public documents that have multiple purposes, including public reassurance, justification of government actions, and information provision, and do not usually contain detailed or complete accounts of actions taken or the reasons for actions and decisions. As a data source they do not convey a complete or definitive picture of government actions. Methods such as interviews, observations and documentary analysis would likely provide a more complex picture of how responses were formulated and implemented, which may shed more light on resilience in the healthcare system.

 Interactions between system levels are also important for understanding resilience, implying the need to focus on how government actions affected other system levels such as organisations, teams and units. Identifying these effects is challenging given the non-linear nature of such interactions and the time over which they may occur. This highlights the need for in depth mixed methods research which can build a picture of the links between actions at the macro level and other system levels and outcomes.

 However, it is not clear which aspects of healthcare systems at the macro level are important in facilitating resilience, or how this could be studied given the difficulty in identifying causal links between actions at various system levels. Studies of adverse incidents and patient harm often identify policy and regulatory failures as contributory factors, but this does not assist in identifying how policy makers and regulators could better support resilience. We need in depth research informed by a systems perspective and using multiple methods to build a better picture of resilience at all system levels.

 Analysis of the system context at the onset of the pandemic could be helpful in understanding how previous decisions constrained resilience when the pandemic occurred. For example, the UK National Health Service, despite being highly rated for safety and efficiency, had low numbers of intensive care unit beds compared to comparable nations,^14^ a longstanding shortage of nursing and medical staff,^15^ and had experienced a decade of austerity that reduced budgets and led to degraded public health infrastructure and leadership.^16^ There had been intense political focus on Brexit during 2019 culminating in a change of government and withdrawal from the European Union in early 2020. This large structural and social change was widely believed to have the potential to exacerbate staffing shortages even without a global pandemic which created the need for a rapid increase in staffing. Understanding how these pressures may have contributed to reducing the margin of manoeuvrability and the capacity to adapt during the pandemic is important and will require in depth mixed methods research to understand resilience between system levels, across time and space.

## Conclusion

 The research challenges of understanding dynamic complex systems in which resilience operates at multiple levels and scales of action are considerable, but necessary for the evolution of the field. Smaggus and colleagues’ paper contributes insight into healthcare system resilience in two jurisdictions and highlights how RHC theory can assist with analysing and understanding the responses. It provides a thoughtful analysis to inform the RHC evidence base, and policy-makers, and on which to build future research. Challenges for the field include the development of a parsimonious, integrated multilevel resilience framework, detailed analysis of the macro level context and its relationship to system resilience, and the development of research methods and designs that are capable of identifying complex, multi layered interactions between system levels. Given the recent experience of the COVID-19 pandemic this is an urgent need and should motivate further efforts to create RHC systems.

## Ethical issues

 Not applicable.

## Competing interests

 Author declares that she has no competing interests.

## Author’s contribution

 JEA is the single author of the paper.
